# Do We Need a Human *post mortem* Whole-Brain Anatomical Ground Truth in *in vivo* Magnetic Resonance Imaging?

**DOI:** 10.3389/fnana.2018.00110

**Published:** 2018-12-05

**Authors:** Anneke Alkemade, Josephine M. Groot, Birte U. Forstmann

**Affiliations:** Integrative Model-Based Cognitive Neuroscience Research Unit, University of Amsterdam, Amsterdam, Netherlands

**Keywords:** *post mortem* neuroanatomy, non-invasive neuroimaging, spatial distortions, 7 Tesla MRI, subcortex

## Abstract

Non-invasive *in vivo* neuroimaging techniques provide a wide array of possibilities to study human brain function. A number of approaches are available that improve our understanding of the anatomical location of brain activation patterns, including the development of probabilistic conversion tools to register individual *in vivo* data to population based neuroanatomical templates. Two elegant examples were published by Horn et al. (2017) in which a method was described to warp DBS electrode coordinates, and histological data to MNI-space (Ewert et al., 2017). The conversion of individual brain scans to a standard space is done assuming that individual anatomical scans provide a reliable image of the underlying neuroanatomy. It is unclear to what extent spatial distortions related to tissue properties, or MRI artifacts exist in these scans. Therefore, the question rises whether the anatomical information from the individual scans can be considered a real ground truth. To accommodate the knowledge-gap as a result of limited anatomical information, generative brain models have been developed circumventing these challenges through the application of assumption sets without recourse to any ground truth. We would like to argue that, although these efforts are valuable, the definition of an anatomical ground truth is preferred. Its definition requires a system in which non-invasive approaches can be validated using invasive methods of investigation. We argue that the application of *post mortem* MRI studies in combination with microscopy analyses brings an anatomical ground truth for the human brain within reach, which is of importance for all research within the human *in vivo* neuroimaging field.

## Introduction

In recent years, within the cognitive neurosciences, functional magnetic resonance imaging (fMRI), measuring blood oxygen level dependent (BOLD) signal has become the tool of choice (Bandettini, 2009). The field of non-invasive neuroimaging techniques continues to progress, providing new opportunities with increased spatial and temporal resolution (Bandettini, 2009; De Martino et al., 2017). Registration to average brain templates such as the Montreal Neurological Institute (MNI-) template provide a platform for the creation of an average human brain, as well as an attractive framework in which data from separate studies can be combined, reused and reanalyzed, providing an important contribution to resolving of the reproducibility crisis (Ioannidis, 2005).

In parallel to the ongoing development of non-invasive imaging techniques, the number of clinical autopsies performed is decreasing world-wide (Burton and Underwood, 2007; Shojania and Burton, 2008; Gaensbacher et al., 2012; Blokker et al., 2016). Although these autopsies do not necessarily include the brain, it raises the question whether more classic neuroimaging techniques, in particular *post mortem* microscopy studies can, and should be replaced by modern non-invasive alternatives. *Post mortem* whole-brain atlases based on microscopic dissections, such as the atlases of Schaltenbrand and Wahren (1977), Talairach and Tournoux (1988), Mai et al. (2015), and BigBrain (Amunts et al., 2013) represent laborious investigations of small numbers or even single brains. Combined, these atlases provide information on fewer than 10 whole brains, a number that does not even start to compare to the number of MRI scans that are produced of individual human brains every single day. It is important to acknowledge efforts of several groups world-wide that perform comparisons between MRI with or without the combination with histological data on smaller tissue blocks (e.g., Bürgel et al., 1999; Castellanos et al., 2008; Makris et al., 2013; Adler et al., 2014; Annese et al., 2014; Augustinack et al., 2014; Plantinga et al., 2016). These studies are of great value for understanding of tissue contrast, but are not incorporated in atlasing efforts. Therefore, we conclude that efforts that could potentially provide a *post mortem* ground truth are not funneled back into *in vivo* neuroimaging studies. Confounding factors are an inherent part of human *post mortem* brain research, and include a bias toward old age, potential effects of pharmacological treatment, nutritional status, ante mortem disease state, cause of death, and *post mortem* factors such as the interval between death and fixation, as well as the known effects of the fixative on the shape and MR characteristics of the tissue (Chu et al., 2005; Schmierer et al., 2008; van Duijn et al., 2011; Stüber et al., 2014). Given these limitations and challenges, we would like to discuss the potential value for human *post mortem* brain research in the validation of state-of-the-art non-invasive functional neuroimaging techniques. Such studies could entail efforts to help to determine to what extent structural MRI scans reliably reflect the underlying neuroanatomy, and to what extent structure fits structure?

We would like to initiate the discussion on the value of the definition of a *post mortem* ground truth for *in vivo* functional neuroimaging studies bringing forward four arguments against, as well as four (related) arguments in favor of these efforts. The first two arguments and their counterarguments relate to inherent limitations of working with *post mortem* tissues. Argument 3 and 4 relate to unresolved issues that require additional experimenting and technical developments.

## Argument 1

*Post mortem* studies by definition provide no information on brain function. Anatomical training represents a cornerstone of medical curriculum, and is historically performed on human cadavers. Today’s development in training includes a shift toward using 3D models of anatomical structures (Dirnhofer et al., 2006). Both digital and 3D printed models are of great value, and could gradually replace cadaver training. It can be argued that the lack of insight in the interindividual variability, and function when studying the *post mortem* brain are in sharp contrast with the large number of *in vivo* MRI images that are freely accessible through various data sharing platforms (e.g., NITRC and DataDryad). Studies on BOLD signal have the important advantage that they provide direct information on the activation of specific parts of the brain during specific tasks, allowing to study human brain function in a reliable and detailed fashion (Bandettini, 2009; De Martino et al., 2017).

## Counter Argument 1

A multimodal approach is needed to understand brain function. BOLD MRI signal provides information on blood oxygenation building on the basic assumption that increased neuronal activity results in increased blood flow, and thereby increasing oxygenated hemoglobin, which can be detected using MRI. Like all hemodynamic-based technologies, BOLD MRI measures a surrogate signal, with limited spatial and temporal specificity due to inherent physical and biological limitations (Logothetis, 2008). Interestingly, information on the geometric and topographic characteristics of the vasculature that allow the size estimation of the area from which BOLD signals originates are now available (Turner, 2002). Linking localization of BOLD signal to detailed *post mortem* investigations of the anatomy of the vasculature allows further pinpointing of the anatomical location of the neuronal activity, thereby providing additional anatomical specificity.

Other techniques used to assess brain activity, including invasive micro-electrical recordings (MER), as well as computed tomography (CT) and positron emission tomography (PET) scanning all have their individual benefits, and face their own challenges and limitations. The combined value of individual techniques is recognized within the scientific community, and our as well as other research groups have argued for pipelines to integrate valuable information from complementing research approaches (Logothetis, 2008; Ewert et al., 2017; Forstmann et al., 2017; Horn et al., 2017).

## Argument 2

Not all neuropathological alterations can be detected using *post mortem* investigations. The (potential) value of non-invasive neuroimaging methods for diagnostics is enormous. Dissection of specific brain areas using invasive autopsy procedures inevitably causes damage to brain structures located on the cutting plane. Thereby not allowing complete analyses of the brain. Additionally, it is feasible that fixation procedures can obscure the presence of neuropathological alterations.

## Counter Argument 2

Not all Neuropathological Alterations can be detected using non-invasive neuroimaging. Both qualitative and quantitative diagnostics can greatly benefit from MRI techniques. Probable Alzheimer disease and Parkinson’s disease can be diagnosed in the clinic, and non-invasive imaging techniques play an important role (Jack et al., 2011; Heim et al., 2017). It is evident that we can visualize neuropathology, but at the same time it is generally accepted that not all abnormalities are visible on non-invasive scans, and misdiagnosis of neuropathological conditions using MRI scans can occur (Kloppel et al., 2008). A probable diagnosis of these neurodegenerative diseases, which fits the clinical definition, but can only be confirmed after death using histological approaches. Through the histological analyses, it also becomes clear which smaller pathological changes cannot be detected. This is of grave importance for diagnostic and research purposes in early stage disease, and for the study of disease progression.

## Argument 3

Individual anatomical scans can provide an anatomical ground truth. Structural MRI scans acquired for fMRI studies are undergoing continuous development and signal to noise ratios have been shown to improve proportional to the applied field strength (Chandran et al., 2016). Additionally, development of novel contrasts tailored to the specific tissue properties of individual brain structures, such as their iron content, contribute to improved visualization of small subcortical nuclei for, e.g., the subthalamic nucleus (Ogg et al., 1999; Rauscher et al., 2005; Manova et al., 2009; Schafer et al., 2009; Vertinsky et al., 2009; Alkemade et al., 2017). At the same time progress is being made to improve white matter imaging and tractography (Maier-Hein et al., 2017). The impressive and constantly improving quality of *in vivo* MRI scans has the potential to increasingly replace *post mortem* investigations.

## Counter Argument 3

The ground truth provided by individual anatomical scans requires testing. At present, we cannot exclude that MRI techniques may suffer from undefined spatial distortions in the living brain. Therefore, the reliability of the assumed ground truth provided by individual MRI images requires testing. It is unclear to what extent spatial distortions exist in anatomical scans, due to tissue or physiological properties, or MR artifacts. Therefore, the information provided by the individual scans can be considered a surrogate ground truth at best. Horn and other researchers have therefore developed methods to accommodate the knowledge-gap, and created generative brain models circumventing these challenges through the application of assumption sets without recourse to any ground truth or individual scan (Horn et al., 2017). Interestingly, this technique also allows the warping of histological data into MNI-space (Ewert et al., 2017). Even though these efforts are valuable, in our opinion closing of the knowledge-gap, and the definition of an anatomical ground truth without potential distortions is preferred. Definition of such a ground truth requires a system in which non-invasive approaches can be validated using invasive methods of investigation. This requires the application of *post mortem* MRI studies followed by histological validation of the images in the same tissue specimens, through which potential distortions can be assessed. Valuable progress is being made in this field, including the application of polarized light imaging, which allows detailed imaging of white matter and its directionality in microscopy analyses (Palm et al., 2010). Additionally, techniques are being developed for registration of results obtained in dissected tissue to MRI space (Zemmoura et al., 2014, 2016).

## Argument 4

**FIGURE 1:**
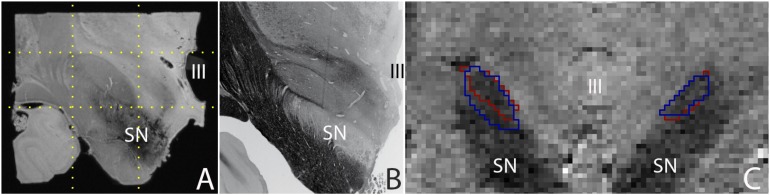
**(A)** 0.06 mm isotropic MRI image of a human *post mortem* subthalamic nucleus (one hemisphere). The STN is located within the square indicated by the dotted lines (adapted from Weiss et al., 2015), **(B)** Histological preparation of the STN (adapted from Mai et al., 2015), and **(C)** 0.8 mm isotropic MRI image of an *in vivo* subthalamic nucleus (left and right hemisphere). Outlines indicate the location of the structure as assessed by two independent raters (adapted from Alkemade et al., 2017). SN, substantia nigra; III, third ventricle.

*Post mortem* MRI studies provide unchallenged spatial resolution and contrast. State of the art *in vivo* MRI studies provide a high level of anatomical detail, which will most likely continue to improve in the future. Today’s high quality images allow the parcellation of a number of subcortical brain nuclei, including the subthalamic nucleus (STN), which is a target for deep brain stimulation (DBS) surgery (Benabid et al., 2009). Using *post mortem* tissue specimens, we have been able to obtain a 60 μm isotropic resolution image of the STN using ultra-high field MRI scanning, in which all borders of the STN are clearly discernible, providing a higher level of anatomical detail than can be obtained *in vivo*. (Figure 1; Weiss et al., 2015). It can be argued that *in vivo* MRI scanning techniques will improve further, and motion correction will allow to compensate for movement artifacts, allowing for improved *in vivo* scan resolutions which will potentially approach the level of detail that can be obtained with *post mortem* MRI scans for subcortical nuclei as well as smaller fiber bundles.

## Counter Argument 4

A majority of subcortical nuclei, as well as smaller fiber bundles still cannot be visualized using (*post mortem*) MRI. Indistinguishable brain structures include a subset of individual thalamic, and hypothalamic nuclei, among which a number of potential DBS targets. As a result, any (functional) MRI signal ascribed to these structures can only be based on anatomical orientation using landmarks. To what extent orientation based on anatomical landmarks is reliable, requires further confirmation using *post mortem* microscopy sectioning approaches, and cannot be achieved using a ground truth based on *post mortem* MRI. Additionally, given the high spatial resolution that can be obtained *post mortem* (see Figure 1), such an approach also has the potential to provide more detailed information on the interindividual variation by more accurate delineations of the borders of individual brain structures. It is important to note that *post mortem* scans registered to MNI-space have been subject to the same registration limitations as present *in vivo*. Additionally, individual specimens do not provide probabilistic information on the location of individual structures. A combined effort of research groups performing *post mortem* MRI scanning and delineating individual brain structures has the potential to create a probabilistic brain atlas that can be registered to MNI-space.

For any neuroscientist using fMRI techniques, as well as for surgeons performing DBS surgery it is important to know the answer to two important questions. The first question is: To what extent are anatomical brain scans representative of the underlying anatomy? Here, we argue that it is possible to answer this question by performing *post mortem* MRI scans, followed by histological validation at the level of the whole brain. The second question is: To what extent are the observations representative for the human brain? Answering this question requires laborious studies on a substantial number of *post mortem* human brain specimens, but is not impossible. Quantitative comparisons to *in vivo* data can subsequently be performed. Quantitative comparisons across modalities could include volume estimates and calculations of differences in the location of individual brain nuclei (Keuken et al., 2014). Challenges to execute the required *post mortem* MRI and histological validation studies are largely logistic. We would like to conclude that *post mortem* MRI and microscopy studies of the human brain will provide an important contribution to the field.

## Author Contributions

All authors were involved in formulating the arguments and in writing the manuscript.

## Conflict of Interest Statement

The authors declare that the research was conducted in the absence of any commercial or financial relationships that could be construed as a potential conflict of interest.
